# Ecological effects of full and partial protection in the crowded Mediterranean Sea: a regional meta-analysis

**DOI:** 10.1038/s41598-017-08850-w

**Published:** 2017-08-21

**Authors:** Sylvaine Giakoumi, Claudia Scianna, Jeremiah Plass-Johnson, Fiorenza Micheli, Kirsten Grorud-Colvert, Pierre Thiriet, Joachim Claudet, Giuseppe Di Carlo, Antonio Di Franco, Steven D. Gaines, José A. García-Charton, Jane Lubchenco, Jessica Reimer, Enric Sala, Paolo Guidetti

**Affiliations:** 1 Université Côte d’Azur, CNRS, FRE 3729 ECOMERS, Parc Valrose, 28 Avenue Valrose, 06108 Nice, France; 2 ARC Centre of Excellence for Environmental Decisions, School of Biological Sciences, The University of Queensland, Brisbane, Queensland Australia; 3 Hopkins Marine Station, Stanford University, Pacific Grove, CA 93950 USA; 4 Oregon State University, 3029 Cordley Hall, Corvallis, OR 97331 USA; 5 Muséum National d’Histoire Naturelle, UMR 7208 BOREA, Station Marine de Dinard - CRESCO, 38 Rue du Port Blanc, 35800 Dinard, France; 6 National Center for Scientific Research, PSL Research University, CRIOBE, USR 3278 CNRS-EPHE-UPVD, Perpignan, France; 7 Laboratoire d’Excellence CORAIL, Moorea, French Polynesia; 8 World Wide Fund for Nature (WWF), Via Po 25/C, 00198 Rome, Italy; 9 Bren School of Environmental Science & Management, University of California, Santa Barbara, CA 93117 USA; 10 Departamento de Ecología e Hidrología, Universidad de Murcia, Campus de Espinardo, 30100 Murcia, Spain; 11 National Geographic Society, Washington, DC 20036 USA; 12 CoNISMa (Interuniversitary Consortium of Marine Sciences), Piazzale Flaminio 9, 00196 Rome, Italy; 13 Centre for Ocean Life, National Institute of Aquatic Resources (DTU-Aqua), Technical University of Denmark, Lyngby, Denmark; 14 Research Unit Biology of Aquatic Organisms and Ecosystems (UMR 7208 BOREA) Sorbonne Universités, MNHN, UPMC, UCN, UA, CNRS, IRD - 43 Rue Cuvier, CP26, 75005 Paris, France; 15 UMS 2006 Patrimoine Naturel - Muséum National d’Histoire Naturelle, CRESCO, 38 Rue du Port Blanc, 35800 Dinard, France

## Abstract

Marine protected areas (MPAs) are a cornerstone of marine conservation. Globally, the number and coverage of MPAs are increasing, but MPA implementation lags in many human-dominated regions. In areas with intense competition for space and resources, evaluation of the effects of MPAs is crucial to inform decisions. In the human-dominated Mediterranean Sea, fully protected areas occupy only 0.04% of its surface. We evaluated the impacts of full and partial protection on biomass and density of fish assemblages, some commercially important fishes, and sea urchins in 24 Mediterranean MPAs. We explored the relationships between the level of protection and MPA size, age, and enforcement. Results revealed significant positive effects of protection for fisheries target species and negative effects for urchins as their predators benefited from protection. Full protection provided stronger effects than partial protection. Benefits of full protection for fish biomass were only correlated with the level of MPA enforcement; fish density was higher in older, better enforced, and —interestingly— smaller MPAs. Our finding that even small, well-enforced, fully protected areas can have significant ecological effects is encouraging for “crowded” marine environments. However, more data are needed to evaluate sufficient MPA sizes for protecting populations of species with varying mobility levels.

## Introduction

Marine protected areas (MPAs) have emerged as a prominent management tool for the conservation and recovery of marine ecosystems and their ecosystem services^1^. As of 2015, announced and implemented MPAs around the world covered 3.6% of the ocean^2^, whereas actively managed MPAs covered only 2.1%^3^. The level of protection in MPAs varies from fully protected, where all extractive activities are prohibited, to partially protected, where some extractive activities are allowed but with varying restrictions^2, 4^. Over the past decade, many countries have begun to establish large fully protected MPAs to protect biodiversity, provide greater resilience to climate change, and achieve global conservation targets such as the Convention on Biological Diversity’s Aichi Target 11, which aims to protect at least 10% of the ocean in MPAs by 2020. Most of the large fully protected MPAs are in remote locations with relatively low human density and impacts^5^. Establishing and implementing MPAs in heavily human-dominated regions is a greater challenge, because multiple users often have conflicting interests and MPAs may be considered as obstacles to their activities^6, 7^.

The Mediterranean Sea is a densely populated region where multiple human activities have placed stress on biodiversity, food webs, and ecosystems for centuries^8–10^. The coastal region of the Mediterranean Sea is home to more than 150 million people and is by far the largest global tourism destination, attracting almost a third of the world’s international tourists annually (343 million out of 980 million worldwide in 2014, with a projection of 500 million by 2030)^11^. Consequently, the demand for marine resources and space is very high, and users often oppose the establishment of MPAs, which may limit or displace their activities (e.g., local commercial and recreational fishing, boating, diving). In such contexts, understanding if MPAs are effective and under what circumstances is essential to raising public and decision-maker awareness and informing decisions about creation, maintenance, expansion, management, enforcement and support for MPAs.

The ecological effects of fully protected areas (also called ‘no-take areas’ or ‘marine reserves’) have been widely documented in both temperate and tropical regions, and this information has often been synthesized in regional and global studies^12–20^. Observed ecological effects include increases in sizes of organisms, density and biomass of commercially exploited species and whole assemblages, reproductive potential, species richness, live cover of benthic organisms, and restoration of trophic interactions (e.g. Selig and Bruno^18^, Edgar *et al*.^20^, Guidetti and Sala^21^ and Floros *et al*.)^22^. In contrast, information on the responses of organisms, populations and communities to partial protection has been synthesized less often in meta-analyses (but see Lester and Halpern^23^, Sciberras *et al*.^24^ and Sala and Giakoumi^25^, and the ecological effectiveness of partially protected areas has often been questioned (e.g. Costello and Ballantine)^26^. Because many MPAs around the world are multiple-use areas that include partially protected areas, especially in densely populated regions, it is important to assess whether partial protection affects species and communities and to what extent.

Species that benefit from protection, particularly large-bodied, slow-growing and late-reproducing predators, are expected to be more abundant and larger in older and larger fully protected MPAs^27–30^. In practice, increases in fish biomass and density have been found to be positively correlated with the age and size of MPAs in some cases (e.g. Claudet *et al*.^14^), but this is contradicted in other studies^31, 32^. One factor that seems to be consistently positively correlated with fish biomass is the level of MPA enforcement^20, 32, 33^. Identifying which MPA design and implementation characteristics influence the magnitude of the effects of protection is important for guiding MPA policy- and decision-making^34^.

**Figure 1:**
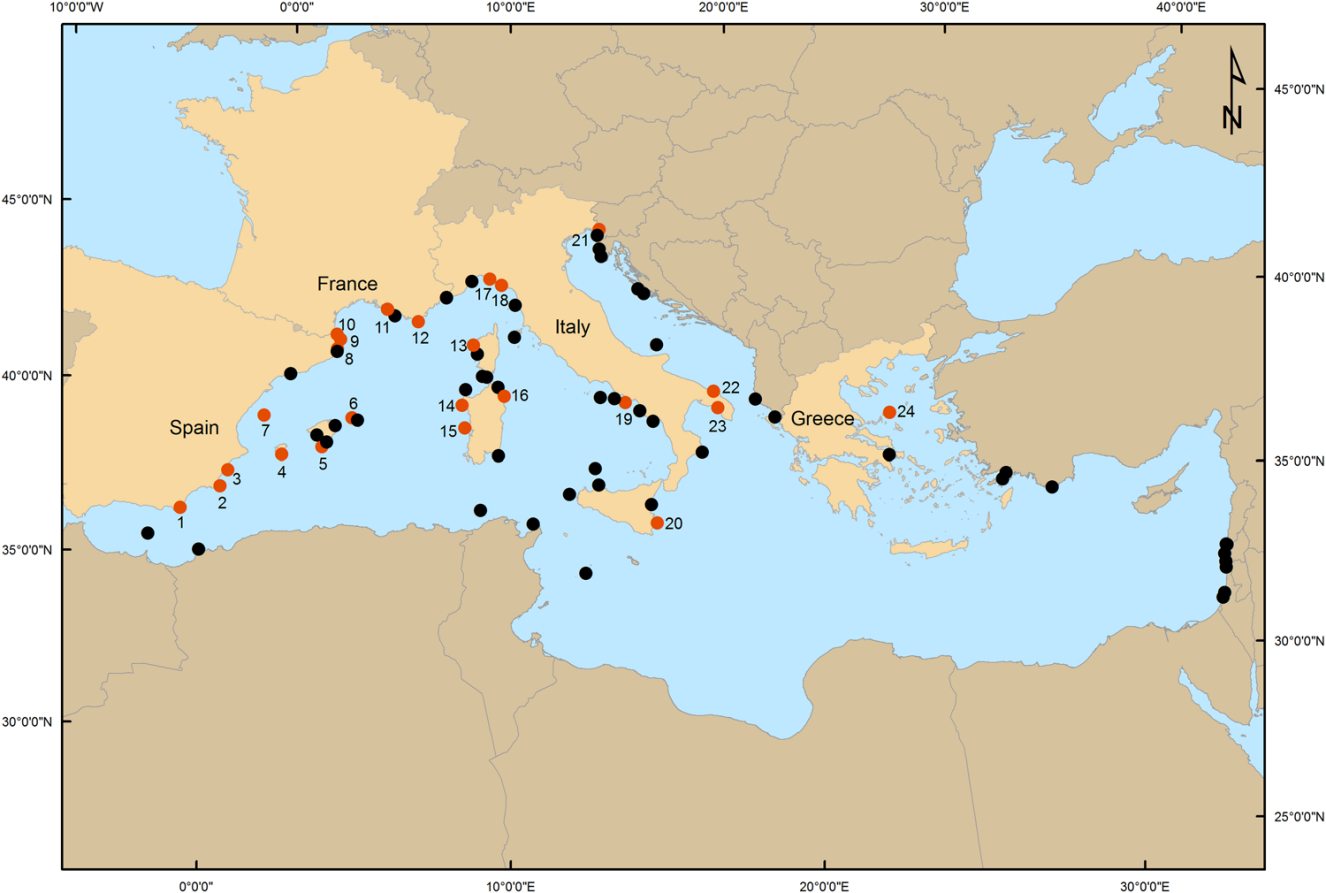
Marine Protected Areas (MPAs) including one or more no-take area(s) across the Mediterranean Sea (76 in total; data source: MAPAMED database)^61^. Orange dots (in highlighted countries) show the 24 MPAs (numbered as in Table 1) for which available data were found through an extensive literature review and that have been included in our analyses. Created by S.G. using ESRI ArcGIS 10.2 Software (http://www.esri.com/software/arcgis).

Currently, only 0.04% of the Mediterranean Sea is fully protected^35^. Typically in the Mediterranean Sea, one or more fully protected (no-take) core areas are surrounded by one or more partially protected (buffer) areas^36^. Here, we present the first meta-analysis focusing on the ecological effects of both fully and partially protected areas within Mediterranean MPAs. We synthesised studies on 24 Mediterranean MPAs (Fig. 1) to assess: (1) the effects of full and partial protection in Mediterranean MPAs on the density and biomass of fish assemblages, commercially and ecologically important fish species with relatively low adult mobility (the dusky grouper *Epinephelus marginatus*, the sea breams *Diplodus sargus sargus* and *D. vulgaris*), and on the density of sea urchins (*Paracentrotus lividus* and *Arbacia lixula*) which are associated with community-wide changes (urchin overgrazing shifts algal forests to barrens)^21^; and (2) the influence of size, age, and level of MPA enforcement on the ecological effects of protection. While our focus is on the Mediterranean region, our results can contribute to a better general understanding of multiple-use MPAs and their management in other densely populated regions of the world.

## Results

**Figure 2:**
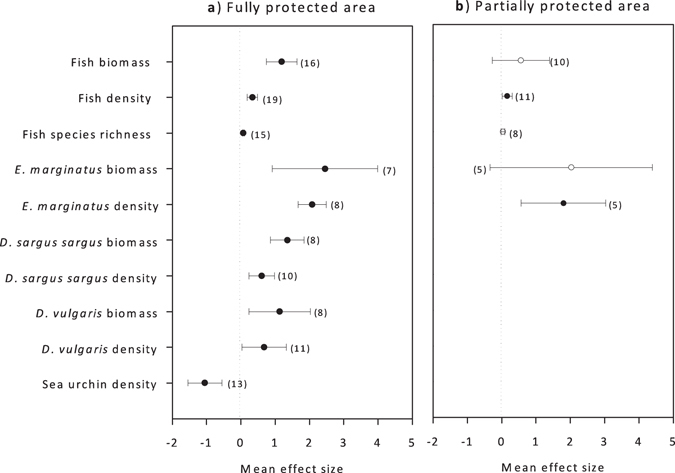
Mean weighted effect sizes in (**a**) fully and (**b**) partially protected areas in Mediterranean MPAs. The graph displays the weighted ratio (*E*) and 95% Confidence Interval (*CI*) in and out (fully or partially) protected areas of: fish assemblage biomass, density, and species richness; dusky grouper (*Epinephelus marginatus*), white seabream (*Diplodus sargus sargus*), and two-banded seabream (*D. vulgaris*) biomass and density; and sea urchin (*Paracentrotus lividus* and *Arbacia lixula*) density. Open dots correspond to mean effect sizes with confidence intervals that overlapped with zero. Sample sizes for each variable are indicated in parentheses next to effect sizes.

The biomass and density of fish assemblages and species of high commercial value were significantly greater in fully protected areas than in unprotected areas nearby (Fig. 2). Total fish biomass and density were on average 2.3 and 1.4 times greater, respectively, in the fully protected areas of the MPAs compared to control fished locations outside MPAs (weighted log-response ratio: E = 1.19 ± 0.45, 95% CI, n = 16; E = 0.34 ± 0.15, 95% CI, n = 19). The strongest effect was on the dusky grouper, *Epinephelus marginatus*, which is a commercially important high-level predator. Dusky grouper mean biomass and density were 10.5 and 7 times greater, respectively, in fully protected areas than in adjacent unprotected sites (E = 2.45 ± 1.53, 95% CI, n = 7; E = 2.08 ± 0.41, 95% CI, n = 8). The mean biomass of two seabreams, *Diplodus sargus sargus* and *D. vulgaris*, was 2.8 and 1.4 times (respectively) greater in fully protected areas than in control sites (E = 1.34 ± 0.50, 95% CI, n = 8; E = 1.13 ± 0.89, 95% CI, n = 8). The effect of full protection on fish species richness was small (E = 0.07 ± 0.03, 95% CI, n = 15), but statistically significant. Sea urchin density was lower in fully protected areas compared to surrounding unprotected areas (E = −1.05 ± 0.53, 95% CI, n = 13). We did not investigate the effect of protection on body size of specific species due to lack of data (see Methods).

There were no differences in total fish biomass and biomass of commercially important species between partially protected and unprotected areas (Fig. 2). However, the mean density of *E. marginatus* was 5 times higher in partially protected areas compared to adjacent fished areas (E = 1.81 ± 1.23, 95% CI, n = 5). Species richness also did not differ between partially protected and unprotected areas.

Full protection was associated with stronger effects than partial protection in all comparisons presented in Fig. 2. For total fish biomass, the effect size of full protection was significantly greater than that of partial protection (t_(5)_ = 2.876, p < 0.05) but not significant for density or diversity (p > 0.1). No statistically significant differences were found between the fully and partially protected areas either in terms of biomass or density for *E*. *marginatus*.

**Figure 3:**
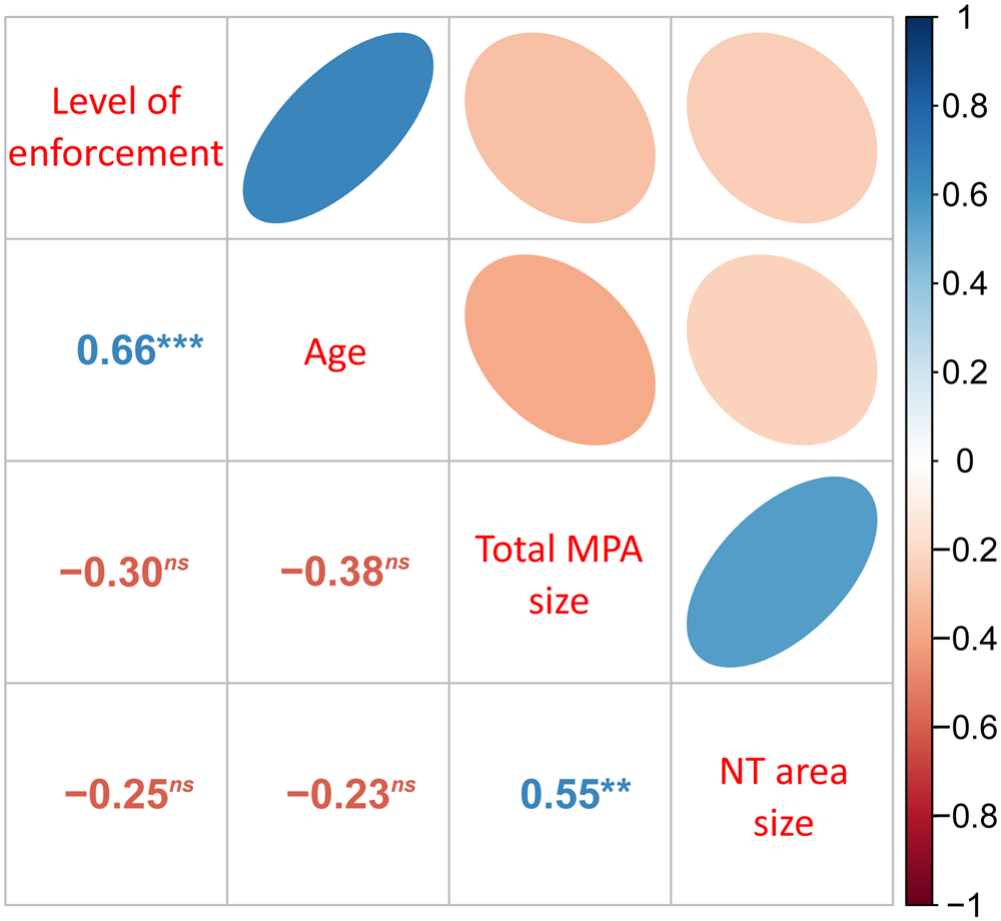
Spearman’s rank correlations among pairs of MPA features. Lower triangular correlation matrix: numbers are Spearman’s rank correlations (rho). Superscripts symbols indicates p-values of Spearman’s rank correlations tests: ^*ns*^p > 0.1; *p < 0.1; **p < 0.01; ***p < 0.001. Upper triangular correlation matrix: Shape and orientation of ellipses are proportional to rho absolute value and direction, respectively.

**Figure 4:**
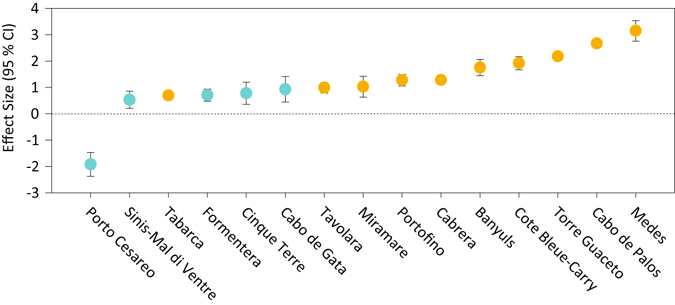
The relationship between mean effect size (95% Confidence Interval) of fish assemblage biomass across the Mediterranean MPAs. Blue dots correspond to MPAs where the enforcement level is low-medium and yellow dots correspond to MPAs where the enforcement level is high (levels of enforcement *sensu* Guidetti *et al*.)^33^.

**Figure 5:**
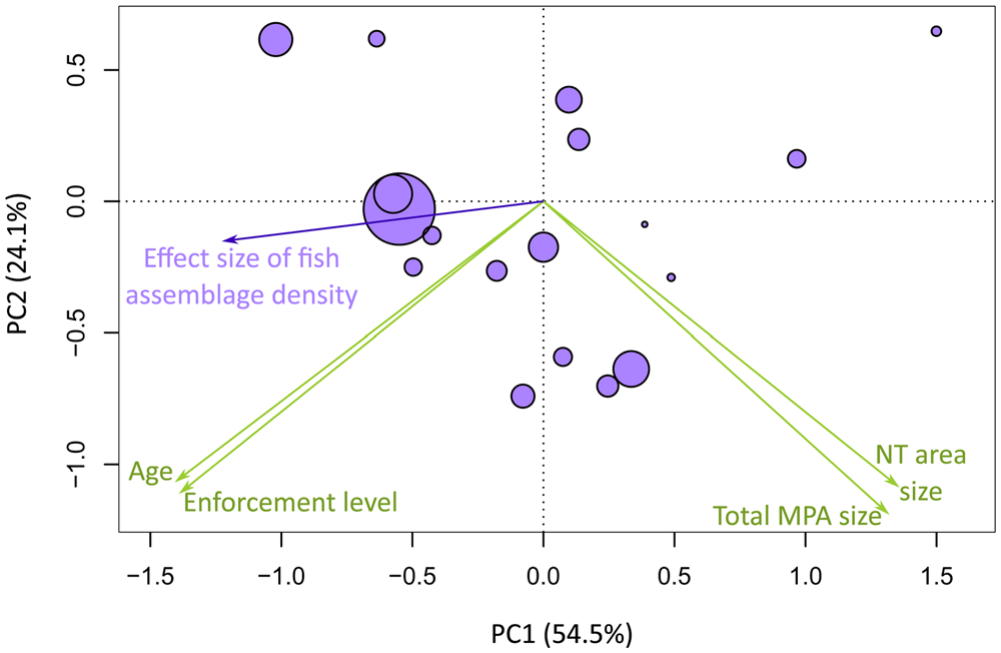
First two axes of the PCA on the four MPA features (green arrows), onto which the effect size of fish assemblage density of each MPA is plotted by using bubbles (bubble size is proportional to effect size value) and a purple arrow (correlation of the response, effect size of fish assemblage density, with PCA axes).

To determine the effects of MPA features (size, age, and enforcement level) on fish assemblage biomass and density in fully protected areas we used weighted linear models (WLM) or weighted generalized linear models (WGLM), depending on the distribution of response variables. We did not run similar analyses for other variables and for partially protected areas because the sample sizes were insufficient (n < 15 studies). MPA features included as predictors in the models were ‘enforcement level’ (2-level factor: low-medium and high), ‘MPA age’ and ‘total MPA size’ (both fully and partially protected areas). The size of the fully protected area was not included because it had a strong, positive correlation with total MPA size (r_s_ = 0.55, p-value of Spearman’s rank correlation test < 0.05) (Fig. 3). It is worth noting that the age and level of enforcement were also correlated (r_s_ = 0.66, Spearman’s: p < 0.05) (Fig. 3); however, both were retained as predictors due to their different nature (continuous vs categorical variable). All other relationships (total size:age, total size:enforcement, no-take area:age and no-take area:enforcement) had negative, marginal correlations (r_s_ between 0.20 and 0.40) (Fig. 3). ANOVAs on all possible WLMs on fish assemblage biomass (see full models in Supplementary Table S2) indicated that only the effect of enforcement level (in the reduced model) was significant (F_(1,13)_ = 7.916, p < 0.05). Highly enforced MPAs hosted the highest fish assemblage biomass (Fig. 4, Supplementary Fig. S1). The variance inflation factors (VIF) for the ANOVA on binomial WGLM of fish assemblage density (Supplementary Table S4) found multi-co-linearity among predictors. In the subsequent principle components regression (PCR; Fig. 5), the effect size of assemblage density had a significant, negative correlation with the first principal component (PC1; Supplementary Table S5), which explained 52.4% of variation among predictors. PC1 was negatively correlated with age and enforcement of MPA and positively correlated with MPA size. This suggested that the effect size of fish assemblage density is higher in older, better enforced, smaller MPAs (Fig. 5).

PCRs on the effect of MPA features on individual fish species and sea urchin variables found that the effect size of *E. marginatus* density was also higher in older, smaller, better enforced MPAs (Supplementary Table S6; Supplementary Fig. S2). The effect size of all other species was not significantly related to age, size, or enforcement of MPAs.

## Discussion

Our results revealed significant positive effects of full protection on fish assemblages, especially on species of high commercial value and relatively low mobility, such as the dusky grouper. Conversely, the mean density of sea urchins was lower in fully protected areas, likely due to the restoration of top-down control by predatory fishes as the latter recovered from exploitation^21, 32^. As the biomass, density, and size of the main predators of adult sea urchins (*D. sargus sargus* and *D. vulgaris*) increase in the fully protected MPA, predation rates on urchins also increase, resulting in a decrease in the biomass, density, and size distribution patterns of the prey (sea urchins)^37^. Large-sized *Diplodus* fishes are much more effective predators than small-sized individuals, capable of preying upon a large spectrum of prey sizes (from juvenile to large-sized sea urchins)^37^. In fished sites, dominated by small- and medium-sized *Diplodus* fishes, large urchins frequently escape from predation pressure as smaller fishes are unable to feed upon them^32, 37^. Nevertheless, the temporal variability of sea urchin density and biomass may be affected not only by predation, but other biological and physical processes, such as recruitment variability and extreme climatic events (see Hereu *et al*.)^38^, acting independently from protection measures.

Partial protection had a positive effect on total fish density and on the density of the dusky grouper. Biomass of fish assemblage and of selected fish species also showed positive trends, but were not statistically significant. Sciberras *et al*.^24^ report significant effects of partial protection on density and biomass of fish assemblages, whereas Lester and Halpern^23^ did not detect significant effects on density or biomass of fish, invertebrates, and algae in partially protected areas. The latter study attributes failure to document such effects to the fact that in most of the studies they reviewed, species of commercial value were targeted in both partially protected areas and unprotected areas. In most partially protected areas in the Mediterranean, both commercial and recreational fishing are allowed, but there are restrictions on gear type (e.g., mesh size of the net) and fishing effort (e.g., number of boats allowed to fish; see Horta e Costa)^4^. The significantly higher mean density of fish assemblages in partially protected areas compared to unprotected areas could be attributed to management measures that limit exploitation, and/or to the spillover of individuals from the fully protected area to the partially protected area^23^. However, commercial and recreational fishing occurring in partially protected areas often target larger individuals of exploited species, and thus the effect of protection on biomass may be reduced^39, 40^. This hypothesis could also explain the significant effect on dusky grouper density, but not biomass, in partially protected areas.

The effect of full protection was significantly higher than the effect of partial protection on fish biomass but not on density, suggesting that large individuals mostly inhabit fully protected areas. Our results differ from previous meta-analyses comparing the effects of full and partial protection. Lester and Halpern^23^ found a significantly greater difference in fully compared to partially protected areas for density but not for biomass, whereas Sciberras *et al*.^24^ found significant differences in both density and biomass. It is important to highlight that the number of partially protected areas and the fishing regulations adopted within each of them vary greatly from one Mediterranean country to another, and even among MPAs within the same country. The number of partially protected zones in Mediterranean MPAs varies from one (e.g. Cerbère-Banyuls, France) to 13 (in the National Marine Park of Alonissos - Northern Sporades, Greece), with each zone having different restrictions for different fishing types (small-scale and/or large-scale commercial and/or recreational fishing). Categorisation of partially protected areas based on the types of fishing activities permitted and their effort potentially explain the high variability in the effect of partial protection detected in this study, especially regarding biomass. When comparing the ecological effectiveness of partially protected areas with different protection categories (based on the IUCN classification scheme) in Canada, Ban *et al*.^41^ found that categories with stricter fisheries regulations were more effective than categories with less restrictions. Nevertheless, responses to protection varied greatly in partially protected areas belonging to the same category. The limited data available on Mediterranean partially protected areas, did not allow us to explore the particular activities that affect differences between full and partial protection.

The effects of full protection on fish biomass were solely associated with the level of enforcement of MPAs, and not the size or age of the MPA. These results are consistent with other studies in the Mediterranean Sea^21, 32^, and further stress the importance of enforcement which has been highlighted as a key feature to achieve economic and social benefits for small-scale fisheries within Mediterranean partially protected MPAs^34^. Yet, it is important to highlight the strong positive relationship we found between the level of enforcement and the age of MPAs. Typically, the enforcement of an MPA in the Mediterranean Sea starts a few years after its designation because there is often a time lag between MPA designation and the availability of funds to support MPA management. Furthermore, compliance is not only linked to surveillance but also to social acceptance. It takes time for ecological benefits to spill-out from MPAs, and hence for local communities to perceive the socio-economic benefits of MPAs. Therefore, older MPAs are more likely to have effective enforcement mechanisms than younger MPAs. Similar confounding effects likely occur in MPAs in other regions of the world but are rarely measured. We also found a weak but significant negative relationship between MPA age and size. Initially, designated MPAs were smaller and their main objective was predominantly biodiversity conservation. More recent MPAs are larger and, in addition to ecological goals, they aim to also promote sustainable socio-economic benefits within their borders. This pattern of implementation also suggests that some effects of MPA size may be masked by their younger age. Decoupling these effects will require more time.

Interestingly, our results suggest that fish density, as well as density of the dusky grouper, is higher in older, better enforced, smaller MPAs. While our finding that higher fish density occurs in older and better enforced MPAs is consistent with studies conducted in the Mediterranean region^21, 32, 33^ and elsewhere^16, 20^, this is not the case for the size of the MPA. Commonly, it is perceived that larger MPAs host higher fish densities per unit area; this perception is also supported by scientific evidence^14, 20^. Our results indicate that in the Mediterranean Sea, even small MPAs (<3000 ha) can be effective (in terms of differences between ‘inside vs. outside’) for some species, when they are fully protected and well-enforced for a sufficient time period that allows the recovery of these species’ local populations. We hypothesize that the following mechanisms cause this result: (1) small MPAs are more likely to be enforced since their surveillance requires relatively fewer resources and may be more easily accepted by local communities; (2) the effects of colonization or migration of commercially targeted species into MPAs (known as ‘spill-in’)^42^ could be more apparent; and (3) they are often sufficiently large to protect the home ranges of species (<10 km^2^) that are primary commercial targets and have relatively low adult mobility^35^. We note that the species used in the analysis have relatively small home ranges, which are approximately the size of the very small fully protected areas in the Mediterranean. Although small, well-enforced MPAs can bring benefits to some economically and socially important species with small home ranges, it is highly unlikely that small MPAs can provide comparable protection for highly mobile species or species with larger home ranges, or that they can restore trophic interactions involving wider-ranging species, or protect the full range of habitats that many species require^2^. Nor are they likely to cause detectable increases in the productivity of fisheries in the wider region.

The MPA features examined in our meta-analyses explained part of the variability in fish communities and one fish species, indicating that the heterogeneity in effect size among MPAs is also influenced by factors not investigated here. These factors could be related to the functional traits of different species^43, 44^, the physical environment, or habitat structure within and outside the MPA^45, 46^, as well as characteristics related to the management and organisational structure of MPAs^47^. However, regardless of the local characteristics that determine the composition of fish assemblages in Mediterranean locales, we can expect structured functional groups within well-enforced fully protected areas, with increases in total fish biomass and dominance of large resident predatory fishes^21^. When interpreting MPA effectiveness, the historical levels of exploitation in the MPAs and the range and relative intensity of exploitation in reference to fished areas should be taken into account as they are two key factors affecting MPA performance^24, 48^. Yet, data on ecological variables before the establishment of MPAs are often lacking (in our review we found only one study) and, thus, the application of proper Before-After-Control-Impact designs is limited (Salomidi *et al*.^49^ but see also Guidetti^50^. A way to overcome this paucity in Before-After studies is to synthesize results of numerous Control-Impact studies and investigate whether the positive effects of protection are consistent under different contexts, as we did herein.

Evidence included in the present study is limited to MPAs in four northern Mediterranean countries: Spain, Italy, France, and Greece. Peer-reviewed scientific information on MPAs from other Mediterranean countries, especially from the southern and eastern sectors, is currently lacking^51^. The paucity of studies reflects both the reality that very few MPAs are implemented within these regions and that results on the effectiveness of implemented MPAs are rarely published in the peer-reviewed literature^32^. Moreover, our analyses were restricted only to fishes and sea urchins, because evidence of the effects of protection in the Mediterranean Sea on other taxonomic groups is scarce. In our literature review, we found studies that provided evidence for positive effects of MPAs on the seagrass *Posidonia oceanica*, the spiny lobster *Palinurus elephas*, the red coral *Corallium rubrum*, amphipods, and gastropods (list of studies in Table S7). The limited data available did not allow us to perform any formal meta-analyses to investigate the effects of MPAs on these taxa at the Mediterranean scale. The scarcity of information on invertebrates and algae, and on all taxonomic groups in some Mediterranean regions, calls for more studies on a broad suite of possible responses to protection and their possible drivers. Obtaining such evidence will allow us to better assess the ecosystem-wide effects of MPAs.

Despite the above limitations, this work presents the first comprehensive and the most updated meta-analysis of Mediterranean MPA studies. In conclusion, our results show positive, general effects of full protection on fish assemblages and key commercial species, as well as general support for predatory control of sea urchin grazers in MPAs. The effects of partial protection in the buffer zones of multiple-use MPAs presented high variability, but overall partial protection was less effective than full protection. Currently, only a tiny proportion (0.04%) of the Mediterranean Sea is reported as fully protected^35^. To achieve effective conservation and recovery of fish populations and marine ecosystems, more MPAs but especially more fully protected areas should be implemented throughout the region. Our finding that even small but well-enforced fully protected areas may provide ecological benefits for harvested species that are not highly mobile is particularly encouraging for human-dominated marine environments such as the Mediterranean Sea, where the establishment of large fully protected areas is challenging. However, the fact that small fully protected MPAs produce benefits inside (based on the ‘inside vs outside’ ratio) doesn’t necessarily mean that they produce significant effects at a population or community level. Individual MPAs should be large enough to protect the home ranges of a large number of species along with a significant fraction of their populations^52, 53^. Nor can this objective necessarily be obtained using networks of small fully protected MPAs. For instance, doubling the biomass inside an MPA or a network of small MPAs but only protecting a very small fraction of the population will produce only a small positive net effect on the species population. Networks of fully protected MPAs have real benefits if a sufficient number of individual MPAs are effectively connected to provide aggregate benefits^62^. The analysis of changes inside MPAs alone is insufficient to address this issue. More studies are necessary to identify the minimum size of individual networked MPAs in the Mediterranean that would be needed to provide population-wide benefits across marine species with different patterns of mobility.

In crowded contexts such as the Mediterranean Sea, the establishment of well-designed networks of well-enforced MPAs, sufficiently large to ensure the persistence of the species by protecting adequate proportions of their populations^35, 53^, involves trade-offs in access that necessarily require a balance between short-term and long-term ecological and socio-economic benefits. Regardless, all species and particularly those with very large home ranges will also require effective fisheries management outside the MPAs to obtain conservation benefits. The results of this analysis demonstrate the need to carefully consider MPA goals and intended outcomes when assigning sizes and protection levels. Positive effects are most likely with full protection, thus the assemblage of MPAs in the Mediterranean may produce greater ecological benefits if existing protection levels were increased.

## Methods

### Data collection

We conducted a comprehensive survey of the peer-reviewed literature to compile a database of studies that investigated ecological effects of MPAs in the Mediterranean Sea. We performed a bibliographic search on Web of Science for the period 1950 to 18/01/2016, using the key words “Marine Protected Area*”, “Marine reserve*”, and “no-take zone*”. These searches were then refined using the term “Mediterranean”. We exclusively considered MPAs in the Mediterranean Sea and thus avoided potential bias associated with combining data from heterogeneous ecoregions. Previous efforts have included Mediterranean MPAs as case studies in meta-analyses conducted at global or continental scales to assess the effects of full protection^12, 13, 15, 16, 54^, or have focused on subregions within the Mediterranean Sea^14, 21^. Moreover, some of these studies used unweighted meta-analytical approaches^15, 21, 54^, which did not consider within-study variability and hence may have not captured the true pattern of the response to protection^55^.

We found a total of 881 papers for which we screened their abstracts to detect studies that measured ecological variables inside (no-take and/or buffer areas) and outside (control sites) the MPA, or before and after MPA establishment. We found 81 peer-reviewed publications that included data both inside and outside MPAs, and only one study quantifying variables before and after the establishment of an MPA. This study was included in analyses. We further narrowed our search within the 81 publications containing data inside and outside MPAs, looking for studies that measured at least one of four key (most commonly measured) variables: density, biomass per area, individual organism size, and/or species richness per area. We retained studies if they explicitly compared (i) a no-take area to an outside area, (ii) a buffer area to an outside area, or (iii) a combination of all three levels of protection (no-take vs. buffer vs. outside). These studies must have quantified the variable(s) and reported a measure of variance (standard deviation or error) from the mean. We found 53 publications that met these criteria. For each study, we extracted data from text, tables, and figures for the four ecological variables inside the no-take area and/or buffer area of the MPA and outside its borders. We extracted data at all available taxonomic levels. If a study reported multiple data points by depth for both MPA and control areas these values were averaged into a single value. If data were available for several habitats (e.g. seagrass meadows and rocky reefs), we used the data for the habitat that the majority of the studies referred to. If studies included multiple time steps, we used the most recent data because they represent the longest duration of protection. If data were not extractable from the figures (e.g. because measure of variance was absent), we directly contacted the authors of the article to request the needed values. To estimate the combined standard deviation (*s*_m_) from multiple datasets we used the following equation:

1 $${s}_{m}=\sqrt{\frac{{\sum }_{\iota =1}^{\kappa }({n}_{i}-1){s}_{i\,}^{2\,}+{n}_{i}{({X}_{i}-{X}_{m})}^{2}}{({\sum }_{i=1}^{k}{n}_{i})-1}}$$

where *n*_*i*_ is the sample size for the dataset *i*, ${s}_{i}^{2}$ its variance, *X*_*i*_ its mean and *X*_*m*_ the combined mean of all (*k*) datasets.

If there were multiple studies on one MPA, we selected only the most recent to use in our meta-analyses to avoid non-independent data. Studies containing data for multiple levels of protection (buffer or no-take areas within MPAs, outside the MPA, or some combination) were preferred over studies only reporting values for no-take areas and outside MPAs even if they were based on less recent data. For example, a study presenting data collected in year 2006 from both no-take and buffer areas of an MPA was preferred over a study presenting data collected in 2008 from only the no-take zone. We excluded studies that considered buffer areas as control sites. This was often the case for studies on Italian MPAs situated on islands, such as the Ustica and Tremiti MPAs, where sampling in areas outside the MPAs was not conducted due to the absence of unprotected areas around the islands.

We also included regional information for fish assemblages^51^ and sea urchins^32^ for MPAs that were not studied at a local level (i.e. comparing data from the MPA and adjacent outside areas). To do this, we extracted information for taxa reported from buffer and no-take areas of MPAs, and compared them with fished areas geographically close to those MPAs. These areas acted as our controls. Data from MPAs and control sites were collected during the same time period. For example Guidetti *et al*.^51^ compared fish density, biomass, and species richness in MPAs (no-take and buffer areas) and fished sites across the Mediterranean Sea. For fish biomass we extracted information for Parc Natural de Cap de Creus, Reserva Marina del Norte de Menorca, Freus de Ibiza y Formentera, and National Marine Park of Alonnisos Northern Sporades. The control for each of these MPAs was the fished area geographically closest to the MPA (in this case, Montgri, Illa de l’Aire, Eivissa, and Ayvalik, respectively). We extracted data on sea urchins from the regional study Sala *et al*.^32^ following the same strategy. Because of the larger spatial scales addressed by these two studies compared to the others, we repeated all analyses with and without these data. Similar results were obtained when these data were included in the meta-analyses, thus they remained in the dataset presented here.

The resulting database contained 24 peer-reviewed publications published between 1988 and 2014 of 24 MPAs in 4 countries (Fig. 1; Supplementary Table S1). For all of these MPAs, we were able to extract sufficient data to estimate the effect of no-take and buffer areas compared to unprotected control sites for biomass, density, and species richness but not for body size of specific species. Fish assemblages (for which biomass, density, and species richness was estimated) presented a similar composition, including all trophic groups, across all studies.

### Predictors of MPA effectiveness

We examined four MPA features as predictors of MPA effectiveness: total MPA size, size of no-take area within the MPA, age of MPA at the time of the survey, and the level of enforcement (Table 1). Information on the year of establishment and size of MPAs was obtained from peer-reviewed papers, grey literature, MAPAMED database^61^, and MPA authorities (official websites and personal communication). The level of enforcement (high, medium, low) was obtained from Guidetti *et al*.^33^ and Sala *et al*.^32^. The relative enforcement category is ‘high’ when poaching is rare and patrol is active and continuous; ‘medium’ when illegal fishing occurs but is limited by infrequent surveillance; and ‘low’ when illegal fishing commonly occurs and surveillance is virtually nonexistent. For the MPAs where published assessments were not available, we consulted five experts for each MPA and retained the most common enforcement ranking for the analyses. Experts were selected by their knowledge of the specific MPAs based on their publication record.

**Table 1:** Features of MPAs included in the meta-analyses. Consult Fig. 1 for MPAs’ exact locations.

Marine Protected Area	Country	Total Surface (ha)	No-take surface (ha)	% No-take of the total surface	Year establishment	Level of Enforcement (Reference)
1. Reserva Marina de Cabo de Gata-Nijar	Spain	12460	816	6.55	1995	Low (Expert judgement)
2. Reserva Marina de Cabo de Palos-Islas Hormigas	Spain	1931	270	13.98	1995	High (Expert judgement)
3. Reserva Marina Isla de Tabarca	Spain	1754	78	4.45	1986	High (Expert judgement)
4. Freus de Ibiza y Fermentera/Freus d’Eivissa i Formentera	Spain	13617	427	3.13	1999	Medium (Expert judgement)
5. Parque Nacional Marítimo - Terrestre del Archipiélago de Cabrera	Spain	8705	360	4.13	1991	High (Sala *et al*.)^32^
6. Reserva Marina del Norte de Menorca	Spain	5119	1111	21.70	1999	Medium (Sala *et al*.)^32^
7. Reserva Marina Islas Columbretes	Spain	5593	1883	33.67	1990	High (Expert judgement)
8. Islas Medas	Spain	511	93	18.20	1990	High (Sala *et al*.)^32^
9. Parc Natural de Cap de Creus	Spain	3056	21	0.69	1998	Medium (Sala *et al*.)^32^
10. Réserve Naturelle de Cerbère-Banyuls	France	650	65	10.00	1990	High (Expert judgement)
11a. Parc Marin de la Côte Bleue - Carry-le-Rouet	France	79460	85	0.11	1982	High (Expert judgement)
11b. Parc Marin de la Côte Bleue - Cap Couronne	France	79460	210	0.26	1993	High (Expert judgement)
12. Parc National de Port-Cros	France	1288	114	8.85	1963	Medium (Expert judgement)
13. Réserve Naturelle de Scandola	France	1000	122	12.20	1975	High (Expert judgement)
14. Area Marina Protetta Capo Caccia-Isola Piana	Italy	2631	38	1.44	2002	Medium (Guidetti *et al*.)^33^
15. Area Marina Protetta Penisola del Sinis-Isola di Mal di Ventre	Italy	26703	374	1.40	1997	Low (Guidetti *et al*.)^33^
16. Area Marina Protetta Tavolara-Punta Coda Cavallo	Italy	15357	535	3.48	1997	High (Sala *et al*.)^32^
17. Area Marina Protetta Portofino	Italy	346	19	5.49	1999	High (Guidetti *et al*.)^33^
18. Area Marina Protetta Cinque Terre	Italy	4554	87	1.91	1997	Medium (Guidetti *et al*.)^33^
19. Area Marina Protetta Punta Campanella	Italy	1539	177	11.50	1997	Low (Guidetti *et al*.)^33^
20. Area Marina Protetta del Plemmirio	Italy	2429	80	3.29	2004	Medium (Expert judgement)
21. Area Marina Protetta Miramare	Italy	30	30	100.00	1986	High (Guidetti *et al*.)^33^
22. Area Marina Protetta Torre Guaceto	Italy	2227	187	8.40	1991	High (Guidetti *et al*.)^33^
23. Area Marina Protetta Porto Cesareo	Italy	16654	173	1.04	1997	Medium (Sala *et al*.)^32^
24. National Marine Park of Alonnisos Northern Sporades	Greece	226000	15439	6.83	1992	Low (Sala *et al*.)^32^

Relationships among the MPA features were explored through bi-plots and Spearman’s rank correlations (r_s_). Before exploring these relationships, the National Marine Park of Alonnisos Northern Sporades was excluded from the analyses as its fully protected area is at least 100 times larger than any other no-take area in the Mediterranean Sea (see Table 1) and at the same time poorly enforced^32^. This made data from the National Marine Park of Alonnisos Northern Sporades a highly influential, yet non-representative outlier. Thus, our dataset for this part of the analysis (investigating the predictors of MPA effectiveness) included 23 studies. For Cabo de Palos, Medes and Tabarca different studies (conducted in different years) were used to detect the response of different ecological variables to protection. For these three sites, data on MPA age (which was measured extracting the date of the survey from the date of the MPA establishment) were averaged to explore relationships among reserve features.

### Meta-analyses

#### Effect size estimation

To quantify the effect of protection, we estimated the effect size (*e*) for each study by using the log-response ratio ln R^56^, calculated as ln (*X*_*T*_ */X*_*c*_), where *X*_*T*_ and *X*_*c*_ are the mean values of a variable (biomass, density, or species richness) inside (treatment site) and outside the MPA (control site), respectively. The variance associated with the effect size (*v*_*e*_) is:

2 $$ve=\frac{{S}_{T}^{2}}{{N}_{(T)}{X}_{T}^{2}}+\frac{{S}_{C}^{2}}{{N}_{(C)}{X}_{C}^{2}}$$

where *S* ^*2*^_*T*_ and *N*_*T*_ are the variance and sample size of the variable estimated inside the MPA and *S* ^*2*^_*C*_ and *N*_*C*_ the variance and sample size outside the MPA.

We generated effect sizes for fish assemblages (biomass, density, species richness); a set of high-value commercial fish species, the dusky grouper *Epinephelus marginatus*, the white seabream *Diplodus sargus sargus* and the two-banded seabream *D. vulgaris* (biomass and density); and two sea urchin species, *Paracentrotus lividus* and *Arbacia lixula* (density) in each MPA. Effect sizes for fish assemblages were estimated both for fully protected vs. fished areas and for partially protected vs. fished areas.

Effect sizes were weighted to ensure greater contribution of the most robust studies^55^. We used the inverse of the sum of the within study variances (*w*_*i*_) with the among-study variance:

3 $${w}_{i}=\frac{1}{{v}_{{e}_{i}}}$$

The overall effect size (*E*) was calculated as:

4 $$E=\,\frac{{\sum }_{i=1}^{n}{w}_{i}{e}_{i}}{{\sum }_{i=1}^{n}{w}_{i}}$$

The variance of *E* was calculated as

5 $${S}_{E}^{2}=\frac{1}{{\sum }_{i=1}^{n}{w}_{i}}$$

and its 95% confidence interval as

6 $$CI=E\pm {t}_{a/2(n-1)}\ast {S}_{E}$$

where *t* is a critical value determined from the *t*_*n*−*1*_ distribution. The effect sizes were considered to be significantly different from zero if their confidence interval did not overlap zero.

To detect whether there was a significant heterogeneity in the effect sizes, we calculated the total heterogeneity (*Q*_*T*_)

7 $${Q}_{T}=\,\sum _{i=1}^{n}{w}_{i}\,{({e}_{i}-E)}^{2}$$

Q_T_ was tested against a χ^2^ distribution with n − 1 degrees of freedom. A significant deviation from the null hypothesis that all *e*_*i*_ are equal indicated that there is potentially some structure in the data. This means that our samples (studies) do not come from a homogenous pool and that the variability between studies can be attributed to factors other than chance alone (e.g. variation in MPA features)^57^.

We, therefore, built mixed-effects models to account for random variation as well as the variation within and between studies. We calculated an estimate of the pooled study variance

8 $${\sigma }_{pooled\,}^{2}=\frac{{Q}_{T}-(n-1)}{{\sum }_{i=1}^{n}{w}_{i}-\,\frac{{\sum }_{i=1}^{n}{w}_{i}^{2}}{{\sum }_{i=1}^{n}{w}_{i}}}$$

And weights for the mixed effects model were calculated as

9 $${w}_{i(rand)}=\frac{1}{{v}_{i}+{\sigma }_{pooled}^{2}}$$

and re-calculated the overall effect size (*E*) and associated 95% confidence interval using ${w}_{i(rand)}$.

The mean values and confidence intervals of effect sizes estimated by the mixed-effects models were used for further analyses and are presented in the result section (Figs 2 to 5).

#### Effects of MPAs features

For dependent variables with an effect size significantly different from zero and heterogeneous (Q_T_, p < 0.05), we explored possible relationships between the effect size and the MPA features (age, total surface area, no-take area, and level of enforcement; Table 1) using two different approaches depending on the sample size (n).

#### *First approach for n* ≥ 15

When sample size was reasonably large (n ≥ 15), weighted linear models (WLM) or weighted generalized linear models (WGLM) were applied depending on the distribution of the response variable being considered. We used the same weights that were calculated for our mixed-effects models, which account for variation within and between studies. The MPA feature ‘no-take area surface’ was not included to reduce model complexity while accounting for the small sample size. No-take area was highly correlated with total area (Spearman rank’s correlation r_s_ = 0.55), thus most of the information was conserved. Total area was log-transformed due to a right-skew. To reduce the number of estimated coefficients and to better balance the statistical design, the categorical predictor “enforcement” was coded with two levels, low-medium and high. Low and medium were pooled because they were less represented in the dataset when compared to high (Table 1). No studies shared MPAs, therefore all studies were independent and no random study factor was included. Full models, including all possible interactions among predictors, were fitted and ANOVA was used to assess significance of terms. Highly non-significant terms (p > 0.25) were pooled into the residuals to increase the power of significance tests on the remaining terms (Supplementary Text S1). Variance Inflation Factor (VIF) was then used to assess multicollinearity among terms, which may confound ANOVA results. When VIF indicated multicollinearity, Principal Component Regression (PCR) was used as an alternative to W(G)LM. The four MPA features were used to build the principal components because multi-co-linearity is acceptable within the PCR framework. In PCR, the factor “enforcement” was coded as a numerical variable for each level: “Low” = 1, “Medium” = 2, and “High” = 3 (as opposed to the W(G)LMs, where enforcement was treated as a categorical variable). Only Principal Component (PC) 1 and PC2 (explaining > 50% of MPA features variations) were retained and used as predictors in new W(G)LMs. ANOVA was again used for testing relationships between the response variable and the principal components.

#### *Second approach for n* < 15

When the sample size was small (n < 15) for using W(G)LMs with MPA features as predictors (i.e. full model containing 7 terms), we performed PCR as described above.

### Comparison of effect sizes of full and partial protection

To examine the differences in effect size between full and partial protection, we performed pairwise t-test comparisons for each variable.

### Software used

During the stage of data collection, we used software WebPlotDigitizer (http://arohatgi.info/WebPlotDigitizer/app/) to extract data from publications that were presented in figures. Analyses exploring the relationships between the effect sizes of variables and MPA features were performed in the R environment^58^ using the packages ‘base’, ‘vegan’^59^ and ‘MASS’^60^.

### Data availability

All sources of data analysed in this meta-analysis are reported in the Supplementary Information file.

## Electronic supplementary material

Supplementary Online Material
