# Author Correction: Ecological effects of full and partial protection in the crowded Mediterranean Sea: a regional meta-analysis

**DOI:** 10.1038/s41598-018-36021-y

**Published:** 2018-11-30

**Authors:** Sylvaine Giakoumi, Claudia Scianna, Jeremiah Plass-Johnson, Fiorenza Micheli, Kirsten Grorud-Colvert, Pierre Thiriet, Joachim Claudet, Giuseppe Di Carlo, Antonio Di Franco, Steven D. Gaines, José A. García-Charton, Jane Lubchenco, Jessica Reimer, Enric Sala, Paolo Guidetti

**Affiliations:** 1 Université Côte d’Azur, CNRS, FRE 3729 ECOMERS, Parc Valrose, 28 Avenue Valrose, 06108 Nice, France; 2 ARC Centre of Excellence for Environmental Decisions, School of Biological Sciences, The University of Queensland, Brisbane, Queensland Australia; 3 Hopkins Marine Station, Stanford University, Pacific Grove, CA 93950 USA; 4 Oregon State University, 3029 Cordley Hall, Corvallis, OR 97331 USA; 5 Muséum National d’Histoire Naturelle, UMR 7208 BOREA, Station Marine de Dinard - CRESCO, 38 Rue du Port Blanc, 35800 Dinard, France; 6 National Center for Scientific Research, PSL Research University, CRIOBE, USR 3278 CNRS-EPHE-UPVD, Perpignan, France; 7 Laboratoire d’Excellence CORAIL, Moorea, French Polynesia; 8 World Wide Fund for Nature (WWF), Via Po 25/C, 00198 Rome, Italy; 9 Bren School of Environmental Science & Management, University of California, Santa Barbara, CA 93117 USA; 10 Departamento de Ecología e Hidrología, Universidad de Murcia, Campus de Espinardo, 30100 Murcia, Spain; 11 National Geographic Society, Washington, DC 20036 USA; 12 CoNISMa (Interuniversitary Consortium of Marine Sciences), Piazzale Flaminio 9, 00196 Rome, Italy; 13 Centre for Ocean Life, National Institute of Aquatic Resources (DTU-Aqua), Technical University of Denmark, Lyngby, Denmark; 14 Research Unit Biology of Aquatic Organisms and Ecosystems (UMR 7208 BOREA) Sorbonne Universités, MNHN, UPMC, UCN, UA, CNRS, IRD - 43 Rue Cuvier, CP26, 75005 Paris, France; 15 UMS 2006 Patrimoine Naturel - Muséum National d’Histoire Naturelle, CRESCO, 38 Rue du Port Blanc, 35800 Dinard, France

Correction to: *Scientific Reports* 10.1038/s41598-017-08850-w, published online 21 August 2017

This Article contains errors in Reference 7 which was incorrectly given as:

Pascual, M. *et al*. Socioeconomic impacts of marine protected areas in the Mediterranean and Black Seas. **133**, 1–10 (2016).

The correct reference is listed below as reference 1:
