# Supplementary material for: Ecological effects of full and partial protection in the crowded Mediterranean Sea: a regional meta-analysis
Source: Sci Rep. 2017 Aug 21;7:8940. doi: 10.1038/s41598-017-08850-w (PMC5566470; doi:10.1038/s41598-017-08850-w)
Supplement: Supplementary file 1 — Supplementary Online Material [file 41598_2017_8850_MOESM1_ESM.pdf]

Supplementary Online Material

Ecological effects of full and partial protection in the crowded Mediterranean Sea: a regional meta-analysis

Authors: Sylvaine Giakoumi, Claudia Scianna, Jeremiah Plass-Johnson, Fiorenza Micheli, Kirsten Grorud-Colvert, Pierre Thiriet, Joachim Claudet, Giuseppe Di Carlo, Antonio Di Franco, Steve Gaines, Jose Antonio Garcia-Charton, Jane Lubchenco, Jessica Reimer, Enric Sala & Paolo Guidetti

Supplementary Table S1. List of publications from which data were extracted for the meta-analyses.

Bevilacqua, S., Terlizzi, A., Fraschetti, S., Russo G.F & Boero, F. Mitigating human disturbance: can protection influence trajectories of recovery in benthic assemblages?. *J. Anim. Ecol.* **75**, 908-920 (2006).
Ceccherelli, G., Pinna, S. & Sechi, N. Evaluating the effects of protection on *Paracentrotus lividus* distribution in two contrasting habitats. *Estuar. Coast. Shelf. S.* **81**, 59-64 (2009).
Claudet, J., Pelletier, D., Jouvenel, J. Y., Bachet, F.& Galzin, R. Assessing the effects of marine protected area (MPA) on a reef fish assemblage in a northwestern Mediterranean marine reserve: Identifying community-based indicators. *Biol. Conserv.* **130**, 349-369 (2006).
Claudet, J. *et al.* Marine reserves: size and age do matter. *Ecol. Lett.* **11**, 481-489 (2008).
Consoli, P. *et al*. The effects of protection measures on fish assemblage in the Plemmirio marine reserve (Central Mediterranean Sea, Italy): A first assessment 5years after its establishment. *J. Sea Res.***79**, 20-26 (2013).
Di Franco, A. *et al*. Assessing dispersal patterns of fish propagules from an effective Mediterranean marine protected area. *PLoS One.* **7**, e52108 (2012).
Di Franco, A., Di Lorenzo, M. & Guidetti, P. Spatial patterns of density at multiple life stages in protected and fished conditions: An example from a Mediterranean coastal fish. *J. Sea Res.* **76**, 73-81(2013).
Fraschetti, S., Guarnieri, G., Bevilacqua, S., Terlizzi, A. & Boero, F. Protection enhances community and habitat stability: evidence from a Mediterranean Marine Protected Area. *PLoS One*. **8**, e81838 (2013).
García-Charton, J. A.*et al.* Multi-scale spatial heterogeneity, habitat structure, and the effect of marine reserves on Western Mediterranean rocky reef fish assemblages. *Mar. Biol.* **144**, 161-182 (2004).
García-Rubies, A., Hereu, B. & Zabala, M. Long-term recovery patterns and limited spillover of large

predatory fish in a Mediterranean MPA. *PLoS One*. **8**, e73922 (2013).

García-Rubies, A. & Zabala, M. Effects of total fishing prohibition on the rocky fish assemblages of Medes Islands marine reserve(NW Mediterranean). *Sci. Mar.* **54**, 317-328 (1990).

Guidetti, P., Bussotti, S. & Boero, F. Evaluating the effects of protection on fish predators and sea urchins in shallow artificial rocky habitats: a case study in the northern Adriatic Sea. *Mar. Environ. Res.* **59**, 333-348 (2005).

Guidetti, P. *et al.* Italian marine reserve effectiveness: Does enforcement matter?. *Biol. Conserv.* **141**, 699-709 (2008).

Guidetti, P. *et al.* Large-scale assessment of Mediterranean marine protected areas effects on fish assemblages. *PLoS One*. **9**, e91841 (2014).

Harmelin-Vivien, M. *et al.* Gradients of abundance and biomass across reserve boundaries in six Mediterranean marine protected areas: Evidence of fish spillover? *Biol. Conserv.* **141**, 1829-1839 (2008).

Macpherson, E., Gordoa, A. & Garcıa-Rubies, A. Biomass size spectra in littoral fishes in protected and unprotected areas in the NW Mediterranean. *Estuar. Coast. Shelf. S.* **55**, 777-788 (2002).

Prado, P., Farina, S., Tomas, F., Romero, J. & Alcoverro, T. Marine protection and meadow size alter fish herbivory in seagrass ecosystems. *Mar.Ecol-Prog. Ser.***371**, 11-21 (2008).

Sala, E. *et al.* The structure of Mediterranean rocky reef ecosystems across environmental and human gradients, and conservation implications. *PLoS One.***7**, e32742 (2012).

Sahyoun, R. *et al.* Protection effects on Mediterranean fish assemblages associated with different rocky habitats.*J. Mar. Biol. Assoc. U. K.* **93**, 425-435 (2013).

Seytre, C. & Francour, P. A long-term survey of *Posidonia oceanica* fish assemblages in a Mediterranean marine protected area: emphasis on stability and no-take area effectiveness. *Mar. Freshwater Res.* **65**, 244-254 (2014).

Tessier, A. *et al.* Video transects as a complement to underwater visual census to study reserve effect on fish assemblages. *Aquat. Biol.* **18**, 229-241 (2013).

Valle, C. & Bayle-Sempere, J. T. Effects of a marine protected area on fish assemblage associated with *Posidonia oceanica* seagrass beds: temporal and depth variations. *J. Appl. Ichthyol.* **25**, 537-544 (2009).

Villamor, A. & Becerro, M. A. Species, trophic, and functional diversity in marine protected and non-protected areas. *J. Sea Res.* **73**, 109-116 (2012).

Hackradt, C.W. *et al.* Response of rocky reef top predators (Serranidae: Epinephelinae) in and around marine protected areas in the Western Mediterranean Sea. *PLoS One*. **9**, e98206 (2014).

Supplementary Text S1: Method used for detecting terms of W(G)LM to be pooled within residuals, in order to increase the power of ANOVA tests of other terms.

1) The full model (including all main predictors and their interactions) was fit to the data. Weighted linear models (WLM) or Negative Binomial Weighted Generalized Linear Model (WGLM) were used depending on the distribution of the response variable and model residuals.

2) The significance of all terms included in the full model were assessed with analysis of variance (ANOVA) performed with type I sums-of-squares (SS). Because type I SS ANOVA is sensitive to the order of the terms in the model, six ANOVAs were performed exhausting the order of the three main predictors in the saturated model (Table S2 and S3).

3) Terms that were highly non-significant ($p > 0.25$) consistently in the 6 ANOVAs were pooled within residuals.

It should be noted that during the analysis of fish assemblage density at step 1, one site (Medes Island) was removed from the WLM because the log ratio was very high and was an outlier.

Supplementary Table S2. ANOVA tables with type I sums of squares of the six weighted linear models regressing the response assemblage biomass against the predictors Enforced (MPA level of enforcement), Years (MPA age) and Tot (MPA size) and all their interactions (full model). The six models vary in the order the terms are included.

model	term	Df	Sum_Sq	Mean_Sq	F_value	P_value
ABC	Enforced	1	6.587	6.587	5.166	0.057
ABC	Years	1	0.412	0.412	0.323	0.587
ABC	Tot	1	0.012	0.012	0.009	0.927
ABC	Enforced:Years	1	0.165	0.165	0.130	0.730
ABC	Enforced:Tot	1	1.170	1.170	0.918	0.370
ABC	Years:Tot	1	0.042	0.042	0.033	0.861
ABC	Enforced:Years:Tot	1	0.092	0.092	0.072	0.796
ABC	Residuals	7	8.925	1.275	NA	NA
ACB	Enforced	1	6.587	6.587	5.166	0.057
ACB	Tot	1	0.012	0.012	0.009	0.925
ACB	Years	1	0.412	0.412	0.323	0.588
ACB	Enforced:Tot	1	1.116	1.116	0.876	0.381
ACB	Enforced:Years	1	0.219	0.219	0.172	0.691
ACB	Tot:Years	1	0.042	0.042	0.033	0.861

ACB	Enforced:Tot:Years	1	0.092	0.092	0.072	0.796
ACB	Residuals	7	8.925	1.275	NA	NA
BAC	Years	1	1.624	1.624	1.273	0.296
BAC	Enforced	1	5.376	5.376	4.216	0.079
BAC	Tot	1	0.012	0.012	0.009	0.927
BAC	Years:Enforced	1	0.165	0.165	0.130	0.730
BAC	Years:Tot	1	0.428	0.428	0.336	0.581
BAC	Enforced:Tot	1	0.784	0.784	0.615	0.459
BAC	Years:Enforced:Tot	1	0.092	0.092	0.072	0.796
BAC	Residuals	7	8.925	1.275	NA	NA
BCA	Years	1	1.624	1.624	1.273	0.296
BCA	Tot	1	0.692	0.692	0.543	0.485
BCA	Enforced	1	4.695	4.695	3.682	0.096
BCA	Years:Tot	1	0.039	0.039	0.030	0.866
BCA	Years:Enforced	1	0.554	0.554	0.435	0.531
BCA	Tot:Enforced	1	0.784	0.784	0.615	0.459
BCA	Years:Tot:Enforced	1	6.587	6.587	5.166	0.057
BCA	Residuals	7	0.412	0.412	0.323	0.587
CAB	Tot	1	0.012	0.012	0.009	0.927
CAB	Enforced	1	0.165	0.165	0.130	0.730
CAB	Years	1	1.170	1.170	0.918	0.370
CAB	Tot:Enforced	1	0.042	0.042	0.033	0.861
CAB	Tot:Years	1	0.092	0.092	0.072	0.796
CAB	Enforced:Years	1	8.925	1.275	NA	NA
CAB	Tot:Enforced:Years	1	6.587	6.587	5.166	0.057
CAB	Residuals	7	0.012	0.012	0.009	0.925
CBA	Tot	1	0.412	0.412	0.323	0.588
CBA	Years	1	1.116	1.116	0.876	0.381
CBA	Enforced	1	0.219	0.219	0.172	0.691
CBA	Tot:Years	1	0.042	0.042	0.033	0.861
CBA	Tot:Enforced	1	0.092	0.092	0.072	0.796
CBA	Years:Enforced	1	8.925	1.275	NA	NA
CBA	Tot:Years:Enforced	1	1.624	1.624	1.273	0.296
CBA	Residuals	7	5.376	5.376	4.216	0.079

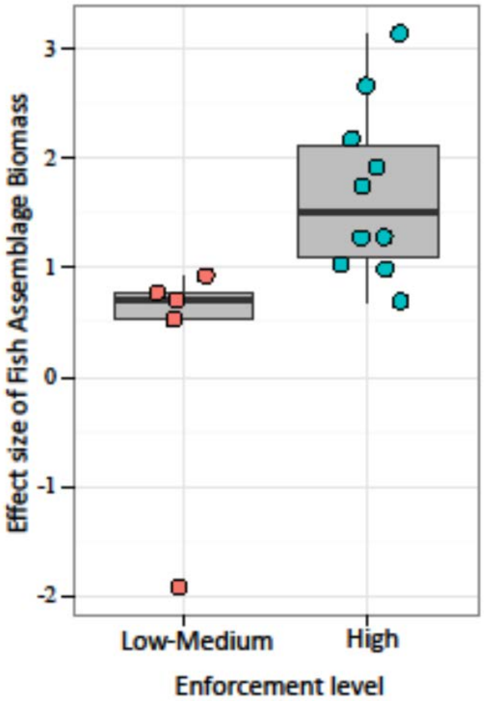

Supplementary Figure S1. Quartiles of effect size of assemblage biomass according to enforcement level.

Supplementary Table S3. ANOVA tables with type I sums of squares of the six saturated negative binomial weighted generalized linear models regressing the response assemblage density against the predictors Enforced (MPA level of enforcement), Years (MPA age) and Tot (MPA size) and all their interactions. The six models vary in the order the terms are included.

model	term	Df	Sum_Sq	Mean_Sq	Chi_sq_value	P_value
ABC	NULL	NA	NA	16	32,7941472	NA
ABC	Enforced	1	6.218	15.000	26.576	0.013
ABC	Years	1	0.026	14.000	26.550	0.872
ABC	Tot	1	1.330	13.000	25.220	0.249
ABC	Enforced:Years	1	0.270	12.000	24.950	0.603
ABC	Enforced:Tot	1	3.739	11.000	21.211	0.053
ABC	Years:Tot	1	2.544	10.000	18.667	0.111
ABC	Enforced:Years:Tot	1	0.032	9.000	18.635	0.858
ACB	NULL	NA	NA	16.000	32.794	NA
ACB	Enforced	1	6.218	15.000	26.576	0.013
ACB	Tot	1	1.339	14.000	25.237	0.247
ACB	Years	1	0.017	13.000	25.220	0.895
ACB	Enforced:Tot	1	3.968	12.000	21.251	0.046

ACB	Enforced:Years	1	0.041	11.000	21.211	0.840
ACB	Tot:Years	1	2.544	10.000	18.667	0.111
ACB	Enforced:Tot:Years	1	0.032	9.000	18.635	0.858
BAC	NULL	NA	NA	16.000	32.794	NA
BAC	Years	1	3.362	15.000	29.433	0.067
BAC	Enforced	1	2.883	14.000	26.550	0.090
BAC	Tot	1	1.330	13.000	25.220	0.249
BAC	Years:Enforced	1	0.270	12.000	24.950	0.603
BAC	Years:Tot	1	0.464	11.000	24.486	0.496
BAC	Enforced:Tot	1	5.819	10.000	18.667	0.016
BAC	Years:Enforced:Tot	1	0.032	9.000	18.635	0.858
BCA	NULL	NA	NA	16.000	32.794	NA
BCA	Years	1	3.362	15.000	29.433	0.067
BCA	Tot	1	2.185	14.000	27.248	0.139
BCA	Enforced	1	2.028	13.000	25.220	0.154
BCA	Years:Tot	1	0.058	12.000	25.162	0.810
BCA	Years:Enforced	1	0.676	11.000	24.486	0.411
BCA	Tot:Enforced	1	5.819	10.000	18.667	0.016
BCA	Years:Tot:Enforced	1	6.218	15.000	26.576	0.013
CAB	NULL	NA	0.026	14.000	26.550	0.872
CAB	Tot	1	1.330	13.000	25.220	0.249
CAB	Enforced	1	0.270	12.000	24.950	0.603
CAB	Years	1	3.739	11.000	21.211	0.053
CAB	Tot:Enforced	1	2.544	10.000	18.667	0.111
CAB	Tot:Years	1	0.032	9.000	18.635	0.858
CAB	Enforced:Years	1	NA	16.000	32.794	NA
CAB	Tot:Enforced:Years	1	6.218	15.000	26.576	0.013
CBA	NULL	NA	1.339	14.000	25.237	0.247
CBA	Tot	1	0.017	13.000	25.220	0.895
CBA	Years	1	3.968	12.000	21.251	0.046
CBA	Enforced	1	0.041	11.000	21.211	0.840
CBA	Tot:Years	1	2.544	10.000	18.667	0.111
CBA	Tot:Enforced	1	0.032	9.000	18.635	0.858
CBA	Years:Enforced	1	NA	16.000	32.794	NA
CBA	Tot:Years:Enforced	1	3.362	15.000	29.433	0.067

Supplementary Table S4. Square root of the variance inflation factor for each term of the negative binomial WGLM with assemblage density as reponse variable, once highly non-

significant terms were pooled within residuals. For each predictor variable, square root of the variance inflation factor equals the number of time the standard error for the coefficient of that predictor variable is larger than what it would be if that predictor variable were uncorrelated with the other predictor variables.

Enforcement	Total area	Age	Enforcement x Total area	Total area x Age
12.126417	7.265279	8.095957	12.609312	9.609482

Supplementary Table S5. ANOVA tables of the negative binomial weighted generalized linear models regressing the response assemblage density against the predictors PC1 and PC2 (see also Figure 5).

	DF	Deviance Residuals	DF residuals	Deviance	
Null			16	32.80	
PC1	1	4.52	15	28.273	**0.03348**
PC2	1	0.25	14	28.025	0.61889

Supplementary Table S6: ANOVA tables of the weighted linear models regressing each response variables at population level, against the predictors PC1 and PC2.

Response variable	term	Df	Sum Sq	Mean Sq	F value	P_Param
DsargDensFull	PC1	1	2.422	2.422	1.744	0.228
DsargDensFull	PC2	1	0.001	0.001	0.001	0.977
DsargDensFull	Res	7	9.719	1.388		
DsargusBioFull	PC1	1	0.568	0.568	0.216	0.662
DsargusBioFull	PC2	1	6.008	6.008	2.284	0.191
DsargusBioFull	Res	5	13.152	2.630		
DvulgBioFull	PC1	1	0.020	0.020	0.034	0.860
DvulgBioFull	PC2	1	0.590	0.590	1.000	0.363
DvulgBioFull	Res	5	2.947	0.589		
DvulgDensFull	PC1	1	0.000	0.000	0.000	0.988
DvulgDensFull	PC2	1	0.000	0.000	0.000	0.995
DvulgDensFull	Res	8	7.151	0.894		
EmarBioBuff	PC1	1	0.083	0.083	0.094	0.789
EmarBioBuff	PC2	1	1.259	1.259	1.425	0.355
EmarBioBuff	Res	2	1.767	0.884		

EmarBioFull	PC1	1	0.711	0.711	0.983	0.378
EmarBioFull	PC2	1	1.379	1.379	1.907	0.239
EmarBioFull	Res	4	2.892	0.723		
EmarDensBuff	PC1	1	0.054	0.054	0.050	0.843
EmarDensBuff	PC2	1	2.234	2.234	2.075	0.286
EmarDensBuff	Res	2	2.153	1.076		
EmarDensFull	PC1	1	6.469	6.469	7.761	0.039
EmarDensFull	PC2	1	0.696	0.696	0.835	0.403
EmarDensFull	Res	5	4.168	0.834		
UrchinDensFull	PC1	1	0.028	0.028	0.031	0.865
UrchinDensFull	PC2	1	0.608	0.608	0.668	0.435
UrchinDensFull	Res	9	8.188	0.910		

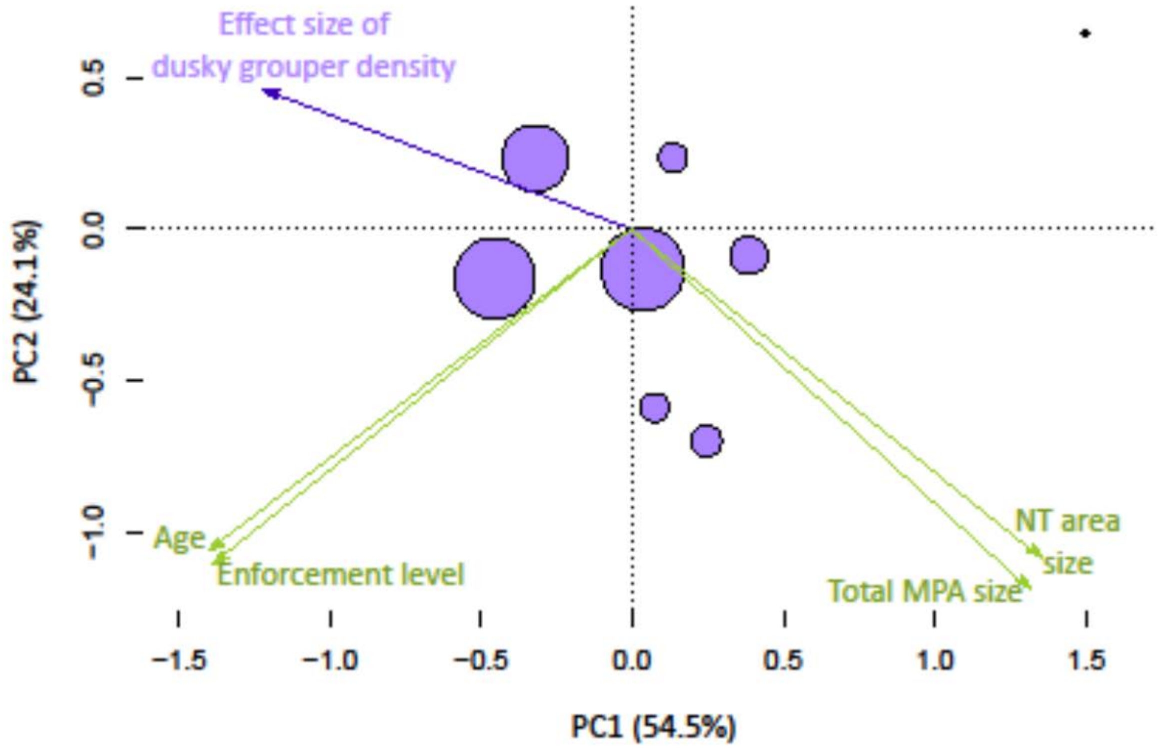

Supplementary Figure S2. First two axes of PCA on the four MPA features (green arrows), onto which the effect size of dusky grouper density of each MPA is plotted by using bubble

(bubble size is proportional to effect size value) and blue arrow (correlation of the response, effect size of dusky grouper density, with PCA axes).

S6.

Supplementary Table S7: List of studies including evidence on the ecological effects of MPAs on taxonomic groups other than fish and sea urchins.

Ferrari, B., Raventos, N., & Planes, S. Assessing effects of fishing prohibition on *Posidonia oceanica* seagrass meadows in the Marine Natural Reserve of Cerbere-Banyuls.*Aquat Bot.* **88,** 295-302 (2008).
Fraschetti, S., Guarnieri, G., Bevilacqua, S., Terlizzi, A., &Boero, F. Protection enhances community and habitat stability: evidence from a Mediterranean Marine Protected Area.*PLoS One.***8,** e81838 (2013).
Goñi, R., Reñones, O., & Quetglas, A. Dynamics of a protected Western Mediterranean population of the European spiny lobster *Palinurus elephas* (Fabricius, 1787) assessed by trap surveys.*Mar Freshwater Res.***52,** 1577-1587 (2001).
Follesa, M.C., et al. Effects of marine reserve protection on spiny lobster (*Palinurus elephas* Fabr., 1787) in a central western Mediterranean area. *Hydrobiologia*. **606,** 63-68 (2008).
Linares, C. et al. Assessing the effectiveness of marine reserves on unsustainably harvested long-lived sessile invertebrates. *Conserv Biol.* **26,** 88-96 (2012).
Sturaro, N. et al. Seagrass amphipod assemblages in a Mediterranean marine protected area: a multiscale approach. *Mar Ecol-Prog Ser.* **506,** 175-192 (2014).
Milazzo, M., Chemello, R., Badalamenti, F., & Riggio, S. Molluscan assemblages associated with photophilic algae in the Marine Reserve of Ustica Island (Lower Tyrrhenian Sea, Italy).*Ital J Zool.* **67,** 287-295 (2000).
Coppa, S. et al. Is the establishment of MPAs enough to preserve endangered intertidal species? The case of *Patella ferruginea* in Mal di Ventre Island (W Sardinia, Italy). *Aquat Conserv.* **26**, 623-638 (2015).
Ferrari, B., Raventos, N., & Planes, S. Assessing effects of fishing prohibition on *Posidonia oceanica* seagrass meadows in the Marine Natural Reserve of Cerbere-Banyuls.*Aquat Bot.* **88,** 295-302 (2008).
